# Draft Genome Sequence of Halomonas sp. KHS3, a Polyaromatic Hydrocarbon-Chemotactic Strain

**DOI:** 10.1128/genomeA.00020-15

**Published:** 2015-03-12

**Authors:** Ana Florencia Gasperotti, Claudia Alicia Studdert, Santiago Revale, María Karina Herrera Seitz

**Affiliations:** a Instituto de Investigaciones Biológicas, Universidad Nacional de Mar del Plata, Buenos Aires, Argentina; b Instituto de Agrobiotecnología Rosario (INDEAR), Rosario, Santa Fe, Argentina

## Abstract

The draft genome sequence of Halomonas sp. KHS3, isolated from seawater from Mar del Plata harbor, is reported. This strain is able to grow using aromatic compounds as a carbon source and shows strong chemotactic response toward these substrates. Genes involved in motility, chemotaxis, and degradation of aromatic hydrocarbons were identified.

## GENOME ANNOUNCEMENT

Bacteria of the genus Halomonas are moderately halophilic microorganisms that belong to the family *Halomonadaceae*, a group of *Gammaproteobacteria*. Microorganisms of this genus have shown a variety of features that make them interesting for biotechnological applications. Concerning bioremediation processes, several Halomonas strains have been involved in biodegradation of aromatic hydrocarbons (1) and other toxic compounds.

Halomonas sp. KHS3 was isolated from hydrocarbon-contaminated seawater of Mar del Plata harbor based on its ability to grow using gas oil as the sole carbon and energy source (2). This strain is a highly motile rod and is strongly chemotactic to phenanthrene and gas oil when analyzed on soft agar plates. Chemotaxis might play a favorable role in biodegradation processes facilitating the accession to the pollutant (3). In order to identify the chemoreceptor(s) responsible for this behavior and get information about the whole chemotaxis system that governs motility in Halomonas sp. KHS3, the genomic sequence was obtained.

To date, there is no information about chemotaxis and its role in bioremediation of xenobiotic compounds for members of the family *Halomonadaceae*.

Genomic DNA of Halomonas sp. KHS3 was isolated and sequenced on an Illumina HiSeq 1500 instrument using 2 × 100-bp reads, resulting in 400-fold genome coverage. The A5-miseq pipeline (4, 5) was used to perform read trimming and correction, contig assembly, crude scaffolding, misassembly correction, and final scaffolding. Twenty-four scaffolds were obtained comprising 5,177,335 bp with an average G+C content of 54.73%. Genome annotation was done using the NCBI Prokaryotic Genomes Annotation Pipeline (PGAP) (6). The RAST annotation server (7) was also used for subsystem classification and functional annotation. A total of 4,493 coding sequences (CDSs) and 68 structural RNAs (60 tRNAs, 8 rRNA) were predicted.

Strain KHS3 was initially placed in genus Halomonas based on the sequence of its 16S RNA gene (Halomonas titanicae strain S6-2-2 and Halomonas sp. MBEE15). The genome sequence showed Chromohalobacter salexigens DSM 3043 and Halomonas elongata DSM2581 as closest neighbors for Halomonas sp. KHS3.

Concerning motility and chemotaxis, several copies of genes coding for some flagellar proteins were identified, as well as other genes involved in flagellar synthesis and rotation.

Halomonas sp. KHS3 has two putative chemotaxis operons containing the basic components found in the E. coli chemotaxis system and a total of 24 chemoreceptors or methyl-accepting chemotaxis proteins (MCPs), similar to the number of MCPs present in other sequenced Halomonas species.

Several genes related to degradation of salicylic and gentisic acids and other aromatic compounds are also present in this genome accounting for the ability of this strain to grow using aromatic hydrocarbons as the sole carbon and energy source. As was previously described for other Halomonas species, several genes related to arsenic resistance were also found. Further studies will be focused on a deeper understanding of chemotaxis signaling in this group of microorganisms and on the interplay between chemotaxis and degradation of xenobiotic compounds.

### Nucleotide sequence accession number.

This whole-genome shotgun project has been deposited at DDBJ/EMBL/GenBank under the accession no. JWHY00000000. The version described in this paper is the first version.
